# Short-term Outcomes in Patients with Carcinoma of the Esophagus and Gastroesophageal Junction Receiving Neoadjuvant Chemotherapy or Chemoradiation before Surgery. A Prospective Study

**DOI:** 10.5041/RMMJ.10339

**Published:** 2019-01-28

**Authors:** Syed Nusrath, Subramanyeshwar Rao Thammineedi, K. V. Vijaya Narsimha Raju, Sujit Chyau Patnaik, Satish Pawar, Ayyagari Santa, Senthil J. Rajappa, Krishna Mohan Mallavarapu, Krishnam Raju, Sudha Murthy

**Affiliations:** 1 Department of Surgical Oncology, Basavatarakam Indo American Cancer Hospital and Research Institute, Hyderabad, India; 2 Department of Medical Oncology, Basavatarakam Indo American Cancer Hospital and Research Institute, Hyderabad, India; 3 Department of Radiation Oncology, Basavatarakam Indo American Cancer Hospital and Research Institute, Hyderabad, India; 4 Department of Laboratory Medicine, Basavatarakam Indo American Cancer Hospital and Research Institute, Hyderabad, India

**Keywords:** Esophagectomy, neoadjuvant chemoradiotherapy, neoadjuvant chemotherapy, resectability

## Abstract

**Background:**

Neoadjuvant chemotherapy (NACT) and neoadjuvant chemoradiotherapy (NACRT) have been demonstrated to improve survival compared to surgery alone in esophageal carcinoma, but the evidence is scarce on which of these therapies is more beneficial, particularly with regard to resectability rates, postoperative morbidity and mortality, and histological responses.

**Objective:**

This study compares the resectability, pathological response rates, and short-term surgical outcomes in patients with carcinoma of the esophagus or gastroesophageal junction receiving NACT or NACRT prior to surgery.

**Methods:**

Patients with resectable carcinoma of the esophagus or gastroesophageal junction adenocarcinoma, squamous cell carcinoma, and adenosquamous histologies were enrolled in this well-matched prospective non-randomized study. Thirty-five patients were given NACT, and 35 NACRT. In the NACT group, 25 patients received three cycles of three-weekly carboplatin and paclitaxel, and 10 received three cycles of cisplatin/5-fluorouracil, while all the patients in the NACRT group received 41.4 Gy of radiotherapy concomitant with five cycles of weekly paclitaxel and carboplatin-based chemotherapy.

**Results:**

Twenty-two patients in the NACT group and 33 patients in NACRT group had resection (*P* value = 0.0027). The percentage of microscopically margin-negative resection (R0 resection) was similar in both the groups (86% versus 88%). The incidences of surgical and non-surgical complications were similar in both the groups (*P=*0.34). There was no 30-day mortality. There was a trend toward more pathological complete regression in the NACRT group (*P=*0.067). The percentage of patients achieving complete tumor regression at the primary site (pT0) was significantly higher in the NACRT group. The down-staging effect on nodal status was similar in both the groups (*P=*0.55). There was a statistically significant reduction in tumor size in the NACRT group. The median numbers of nodes harvested and positive nodes were similar in both the groups.

**Conclusion:**

Patients receiving NACRT had better resectability rates and pathological response rates, but similar postoperative morbidity compared to the NACT group.

## INTRODUCTION

Esophageal and gastroesophageal junctional cancers are aggressive tumors. Although surgery is the prime modality of treatment, cancer recurs in most patients within 2 years after resection, and the patients have a poor median overall survival of 15–18 months.^1^ Moreover, complete resection is not feasible in a significant number of patients owing to the locally advanced nature of the disease, with lymph node metastases being seen almost universally.^1^ Esophagectomy is associated with significant morbidity and mortality.^2^ Furthermore, esophagectomy is a severe physiological trauma on esophageal cancer patients who are often nutritionally depleted, which can delay or compromise the delivery of adjuvant therapy. Hence, neoadjuvant therapy is an attractive option in esophageal cancer where the adjuvant treatment can be completed prior to surgery. The rationale for neoadjuvant chemotherapy (NACT) and neoadjuvant chemoradiotherapy (NACRT) in locally advanced esophageal cancer patients is due to the poor survival rate with surgery alone and the early local and systemic relapses.

Neoadjuvant chemotherapy and NACRT have been demonstrated to improve survival compared to surgery alone, but the evidence is scarce and inadequate regarding which therapy is more beneficial, particularly with regard to resectability rates, postoperative morbidity and mortality, histological tumor response, long-term survival, and health-related quality of life. There is conflicting evidence regarding the effects of perioperative chemotherapy on survival and other outcomes. A recent Cochrane meta-analysis found that perioperative chemotherapy for resectable gastroesophageal adenocarcinoma increases survival compared to surgery alone. In addition, there was a trend for increased survival in patients with gastroesophageal junction tumors as compared to other sites, and in patients who received chemoradiotherapy as compared to chemotherapy in esophageal and gastroesophageal junction tumors.^3^

Esophagectomy is a technically demanding surgery. The overall incidence of postoperative complications rates varies widely between 20% and 80%, and complications can be systemic (e.g. pneumonia, myocardial infarction) and/or specific to the surgical procedure (e.g. anastomotic leaks, recurrent laryngeal nerve injury). Complications occur more frequently after transthoracic surgery than after transhiatal surgery.^1^,^2^

We conducted a prospective well-matched non-randomized study to investigate whether preoperative concurrent chemoradiation is superior to preoperative chemotherapy in terms of resectability and short-term surgical outcomes in carcinomas of the middle, lower esophagus or the gastroesophageal junction in Indian patients.

## MATERIAL AND METHODS

The study was conducted at Basavatarakam Indo American Cancer Hospital and Research Institute, Hyderabad from May 2014 through December 2015. Inclusion criteria were: (1) Histologically confirmed squamous cell carcinoma, adenocarcinoma, and/or adenosquamous carcinoma with potentially resectable middle, lower esophageal, or esophagogastric junction cancer; (2) Clinical stage T2–4a, N0/N+, M0, according to the 7th edition of the American Joint Committee on Cancer Classification (AJCC) tumor–node–metastasis (TNM) classification; (3) Patients aged 18–75 years; (4) Eastern Cooperative Oncology Group (ECOG) performance status score of 1 or lower; (5) Adequate hematologic, renal, hepatic, and pulmonary function; and (6) No history of any other cancer or previous radiotherapy or chemotherapy. The following patients were excluded from the study: Patients with an unresectable tumor invading other adjacent structures (such as the aorta, vertebral body, or trachea [T4b]), metastatic disease (M1), or cervical and upper-third esophageal tumors; and patients who had had previous stomach surgery.

All patients underwent pretreatment staging which included the acquisition of a full patient history, physical examination, complete blood count, liver function test, renal function test, upper gastrointestinal endoscopy with biopsy, computed tomography of the chest and abdomen, and pulmonary function test. Bronchoscopy was done for middle-third esophageal tumors. Chemotherapy and chemoradiation were given as per schedule. A repeat CT scan of the chest and abdomen and an upper gastrointestinal endoscopy were performed 4–6 weeks after completing therapy to assess the patient’s response to therapy and the feasibility of surgery.

Continuous data were expressed as median with range or as mean with standard deviation. Categorical data were compared by Fischer’s exact test and continuous variables by Mann–Whitney’s *U* test, respectively. Data were analyzed according to the intention-to-treat analysis. All statistical comparisons were made with two-tailed tests. A *P* value of less than 0.05 was considered statistically significant. All statistical analyses were performed using statistical software Prism Graph pad version 5 for Windows (GraphPad Software, La Jolla, CA, USA).

### Treatment Protocol

Concurrent chemoradiation was administered to the NACRT group as per CROSS study protocol with weekly intravenous paclitaxel and carboplatin, i.e. on days 1, 8, 15, 22, and 29, with carboplatin targeted at an area under the curve (AUC) of 2 mg per milliliter per minute and paclitaxel at a dose of 50 mg per square meter of body surface area (BSA).^4^ The same chemotherapy drugs were administered in the NACT group (in 25 patients) scheduled in three cycles, three-weekly. The dose of carboplatin was AUC 5–6 and that of paclitaxel was 175 mg/m^2^. Ten patients in the NACT group received three cycles of fluorouracil and cisplatin. These patients belonged to a health scheme that did not approve paclitaxel and carboplatin-based chemotherapy and allowed only for 5-fluorouracil and cisplatin; hence 5-fluorouracil and cisplatin-based chemotherapy was administered to this subgroup. 5-Fluorouracil 800 mg/m^2^ per 24 hours was administered as a continuous infusion on days 1–4 and 29–32. Cisplatin 75 mg/m^2^ was delivered by infusion on day 1 or 2 and again on day 29 or 30. A total radiation dose of 41.4 Gy delivered by a linear accelerator (Elekta Synergy, Elekta Instrument AB, Stockholm, Sweden) was given in 23 fractions of 1.8 Gy each, with five fractions administered per week, starting on the first day of the first chemotherapy cycle. All patients were treated by means of conformal external-beam radiation therapy by CT-based planning. The gross tumor volume (GTV) was defined by the primary tumor and any enlarged regional lymph nodes. The GTV was determined using all available information obtained by physical examination, endoscopy, and CT-thorax/abdomen. The planning target volume (PTV) included a proximal and distal margin of 4 cm; if the tumor extended to the gastric cardia, a distal margin of 3 cm was taken. A radial margin of 1.5 cm around the GTV was taken to include the area of subclinical involvement around the GTV and to compensate for motion artifact.

### Surgery

Patients with resectable disease in both groups underwent surgery within 4–6 weeks after completing therapy. For tumors involving the esophagogastric junction, a laparoscopic-assisted transhiatal resection with D2 lymphadenectomy was preferred. Stomach and esophagus mobilization was performed laparoscopically till the inferior pulmonary ligament level. A small 5 cm supra-umbilical midline incision was made, and the rest of the thoracic esophagus was mobilized by hand. A small left-sided horizontal cervical incision was then made, the medial head of the sternomastoid divided, strap muscles retracted, the esophagus isolated and divided, and the specimen was retrieved through a midline supra-umbilical incision. The specimen was resected with adequate margins, and a gastric conduit tube was constructed (on the right gastroepiploic artery) and transferred to the neck through the posterior mediastinum, and end-to-side esophago-gastric anastomosis was performed using the double-stapled technique. The surgical site was closed after placing a feeding jejunostomy tube (12F Ryles tube) and a hiatal drain. Intercostal chest tubes were placed only when there was a breach of pleura.

Thoracoscopic and laparoscopic-assisted three-stage resection with neck anastomosis (minimal invasive McKeown) with mediastinal lymph node and D2 dissection were performed for tumors in the mid-esophagus and for the few lower-third tumors not accessible by laparoscopy. Mobilization of the thoracic esophagus along with mediastinal lymph node dissection was completed in the semi-prone position, after which the patient position was changed to supine with the rest the of the dissection completed as described above for transhiatal resection.

## RESULTS

From May 2014 through December 2015 a total of 78 patients with esophageal or gastroesophageal junction cancer were screened. Eight patients were excluded from the study, of which five did not meet the inclusion criteria and three did not consent. Hence, 70 eligible patients satisfying the inclusion criteria participated in the study. Thirty-five patients were prospectively assigned to the NACRT group and 35 to the NACT group. In the NACT group 25 patients received three cycles of three-weekly carboplatin and paclitaxel and 10 patients received three cycles of cisplatin/5-fluorouracil, while all the patients in NACRT group received 41.4 Gy of radiotherapy concomitant with five courses of weekly paclitaxel and carboplatin-based chemotherapy. Both of the NACT subgroups (receiving different chemotherapy) had similar demographic profiles. Demographic parameters are shown in Table 1.

**Table 1:** Patient Demographics Parameters.

Demographic Parameter	Neoadjuvant Chemotherapy (NACT, *n*=35)	Neoadjuvant Chemoradiotherapy (NACRT, *n*=35)	*P* value
Median age in years (range)	54 (23–70)	53 (26–70)	0.7199

Gender			
Male	20 (57%)	18 (54%)	0.8106
Female	15 (43%)	17 (46%)	

Median symptom duration in months (range)	2 (0.5–12)	1.25 (0.5–12)	0.4561

ECOG PS score			
0	31 (88.5%)	34 (97%)	0.3565
1	4 (11.5%)	1 (3%)	

BMI (kg/m^2^) (median)	20	24.17	0.0602

Tumor site			
Middle esophagus	5 (14%)	11 (31.4%)	0.2983
Lower esophagus (Siewert I)^*^	12 (32%)	12 (34.2%)	
Esophago-gastric junction (Siewert II)	6 (17%)	3 (8.5%)	
Subcardial (Siewert III)	12 (34.2%)	9 (25.7%)	

Tumor type			
Adenocarcinoma	16 (45.7%)	12 (34%)	0.4451
Squamous cell carcinoma	19 (54.3%)	21 (60%)	
Others	0	2 (6%)	

Tumor in cm			
Median	6	6	0.3656
Range	2.5–8	2.5–8	

Tumor thickness in cm			
Median	1.85	2	0.362
Range	0.6–5.7	0.7–4	

T stage			
Early T stage cT1/2	2 (5.8%)	2 (5.8%)	1
Advanced T stage cT3/4	33 (94.2%)	33 (94.2%)	

Clinical N stage			
Node negative	19 (54.3%)	15 (43%)	0.4734
Node positive	16 (45.7%)	20 (57%)	

Prognostic factors such as sex, age, duration of symptoms, tumor site, tumor type, tumor length, tumor thickness, clinical T and N stage, ECOG performance status (ECOG PS), and body mass index (BMI) were well matched in both groups.

Squamous cell carcinoma constituted 57% (40 of 70 patients) of all cases in both groups. Adenocarcinoma constituted 40% (28 of 70 patients) of all cases in both groups. Median tumor length was 6 cm in both groups; median tumor thickness was 1.85 cm in the NACT group and 2 cm in the NACRT group. Clinical T3 and T4 tumors constituted 94% of cases in both groups. Forty-six percent of the patients in the NACT group and 57% in the NACRT group were node-positive. The median time from the end of therapy to surgery was 7 weeks in the NACT group and 8 weeks in the NACRT group.

Unresectable disease was found in 13 out of 35 patients (37%) in the NACT group. One patient had metastatic disease on post-therapy CT scan, another had a progressive disease with increasing dysphagia and declining performance status, and 11 patients who proceeded to surgery had unresectable locally advanced/metastatic disease at diagnostic laparoscopy precluding curative surgical resection. The remaining 22 patients (63%) had a resectable disease that proceeded to a definitive surgical resection.

In the NACRT group, only two patients (6%) had unresectable disease at diagnostic laparoscopy. The remaining 33 patients (94%) had resectable disease and proceeded to definitive surgery. The percentage of patients with resectable disease was statistically greater in the NACRT group as compared to the NACT group (*P*=0.0027).

### Surgery

Details of surgery is mentioned in Table 2. Twenty-two patients underwent definitive surgery in the NACT group. Six patients underwent minimally invasive transthoracic esophagectomy (TTE), and one underwent open TTE. Thirteen patients underwent minimally invasive transhiatal esophagectomy (THE), and one underwent open THE. One patient with gastroesophageal junction cancer underwent proximal gastrectomy and distal esophagectomy. Thirty-three patients in the NACRT group underwent definitive surgery. Fourteen patients underwent minimally invasive TTE, and one underwent open TTE. Seventeen patients underwent minimally invasive THE, and one underwent open THE.

**Table 2:** Patients Proceeding to Resectable Surgery in Both Groups.

Surgical Procedure	Neoadjuvant Chemotherapy (NACT; *n*=22)	Neoadjuvant Chemoradiotherapy (NACRT; *n*=33)
Transthoracic esophagectomy		
Open	1	1
Minimally invasive	6	14

Transhiatal esophagectomy		
Open	1	1
Minimally invasive	13	17

Open proximal gastrectomy, distal esophagectomy	1	0

### Operative and Clinical Details

The median operative times of 300 minutes in the NACT group and 250 minutes in the NACRT group were not statistically different (Table 3). Median blood loss during surgery was statistically less in the NACRT group compared with the NACT group (200 mL versus 250 mL; *P*=0.0168). Median blood transfusion during hospital stay, median day of extubation, the return of bowel sounds after surgery, the start of jejunostomy feeding and oral feeds, and the passage of stool and flatus were similar in both the groups and are also detailed in Table 3. Median intensive care unit (ICU) stay and hospital stay were also similar in both the groups.

**Table 3:** Operative and Postoperative Details.

Parameters (median)	Neoadjuvant Chemotherapy (NACT; *n*=35)	Neoadjuvant Chemoradiotherapy (NACRT; *n*=35)	*P* value
Operation time (min)	300	250	0.3485
Range	165–490	150–405	

Blood loss (mL)	250	200	0.0168
Range	100–600	50–500	

Blood transfusion	0	0	

Extubation day	0	0	

Bowel sounds (day)	1	1	

Feeding jejunostomy (days)	1	1	

Oral feed (day)	6	6	

Flatus/motion passage (day)	3	3	

Intensive care unit stay (days)	5	4	0.1058
Range	3–10	2–11	

Nasogastric tube removal (day)	4	3	

Drain removal (day)	5.5	6	

Hospital stay (days)	8	8	0.8883
Range	7–18	6–13	

Re-explorations (*n*)	2	0	

### Thirty-day Surgical Morbidity and Mortality

Surgical complications occurring in both the groups are detailed in Table 4. No deaths occurred in either group. In the NACT group, one patient had conduit necrosis, and one had bleeding from the conduit; both underwent repeat exploration. The latter patient also required a tracheostomy. There was one case of recurrent laryngeal nerve palsy (managed conservatively); two cases of cervical esophago-gastric anastomosis leak, which was managed conservatively (including the conduit necrosis case mentioned above); three had cardiac complications; and four experienced pulmonary complications.

**Table 4:** Surgical Complications in Both Groups.

Surgical Complications	Neoadjuvant Chemotherapy (NACT; *n*=22)	Neoadjuvant Chemoradiotherapy (NACRT; *n*=33)
Conduit bleeding	1 (4.5%)	0
Conduit necrosis	1 (4.5%)	0
Recurrent laryngeal nerve palsy	1 (4.5%)	2 (6%)
Chyle leak	0	2 (6%)
Anastomotic leakage	2 (9%)	3 (9%)
Surgical site infection	1 (4.5%)	2 (6%)
Cardiac complications	3 (13.6%)	3 (9%)
Pulmonary complications	4 (18%)	4 (12%)
Death	0	0

In the NACRT group, two patients had recurrent laryngeal nerve palsy (managed conservatively); three had a cervical esophago-gastric anastomosis leak that was managed conservatively; two had a surgical site infection, also managed conservatively; two had a chyle leak (one chylothorax, one chyle abdomen); three had cardiac complications; and four had pulmonary complications. One patient required bronchoscopic lavage for the pulmonary toilet. There were no repeat explorations.

Surgically related complications were graded and classified according to Clavien–Dindo’s methodology.^5^ Higher complication rates (Clavien–Dindo’s grade 3a and greater) versus lower complication rates (Clavien–Dindo’s grade 1 and 2) were similar between both groups (*P*=0.3377).

### R0 Resection and Tumor Regression Grade

Twenty-two patients in the NACT group and 33 patients in the NACRT group proceeded to curative surgery. The percentage of patients who had curative R0 resection was similar in both the groups (86% versus 88%) (Table 5). Three patients (14%) in the NACT group and 15 patients (45.5%) in the NACRT group had complete tumor regression (tumor regression grading [TRG] grade 0) at the primary tumor site; this was statistically significant (*P*=0.019).

**Table 5:** Pathological Parameters in Both Groups.

Parameter	Neoadjuvant Chemotherapy (NACT; *n*=22)	Neoadjuvant Chemoradiotherapy (NACRT; *n*=33)	*P* value
R0 resection	19 (86%)	29 (88%)	1

R1 resection	3 (14%)	4 (12%)	1

Tumor regression grading (TRG) score			0.019
Complete (0)	3	15	
Moderate (1)	1	5	
Minimal (2)	8	9	
Poor (3)	10	4	

TRG 0	3 (14%)	15 (45.5%)	0.019
TRG 1/2/3	19 (86%)	18 (54.5%)	

LVI	10 (45%)	5 (15%)	0.0284

PNI	4 (18%)	6 (18%)	1

Down-staging effect on primary tumor			0.019
pT0	3 (13.6%)	15 (45%)	
pT1/2/3/4	19 (86.4%)	18 (55%)	

Down-staging effect on nodes			0.5533
pN0	15 (68%)	25 (76%)	
pN1/2/3	7 (32%)	8 (24%)	

Pathological complete response	3 (13.6%)	13 (39.4%)	0.0675

Median tumor size (cm)	2.5	0.2	0.0005

No. of lymph nodes resected			0.1872
Median	13	11	
Range	8–32	2–26	

No. of positive lymph nodes			0.5485
Mean	1.72	0.72	
Range	0–11	0–6	

LVI, lymphovascular invasion; PNI, perineural invasion; R0 resection, microscopically margin-negative resection; R1 resection, removal of all macroscopic disease, but microscopic margins are positive for tumor.

## DISCUSSION

Our study did not show any difference in R0 resection rates in patients who underwent resection between the two groups (88% versus 86%). It showed a significant improvement of resectability rate from 63% to 94% with the addition of radiation to paclitaxel and carboplatin-based chemotherapy. There was a tendency to achieve higher complete pathological regression in the NACRT group as compared to the NACT group (*P*=0.0675). The tumor regression score also favored the NACRT group. There was a significant trend to achieve pT0 status, i.e. with complete tumor regression and a TRG score of 0 in the primary tumor site in the NACRT group (*P*=0.0284). However, this tendency was not seen in the nodes. There was a significantly higher proportion of lymphovascular invasion in the NACT group as compared to the NACRT group.

R0 resection has varied widely in different trials. In trials comparing surgery to neoadjuvant chemoradiation, R0 resection ranged from 81% in the Bosset et al. study to 100% in the Lee et al. study in the NACRT group.^6^,^7^ In their surgical group, R0 resection ranged from 69% in the CROSS trial to 95% in the study by Lee et al.^4^,^7^ In NACT, R0 resection was low, ranging from 60% in the MRC trial^8^ to 87% in the FNCLCC trial.^9^ In studies comparing NACT with NACRT, the R0 resection rates in the NACT and NACRT groups varied from 69% and 72%, in Stahl et al.’s study,^10^ to 80.5% and 84.6% in Burmeister et al.’s study, respectively.^11^ In the present study, the R0 resection rate was 86% and 88% in the NACT and NACRT groups, respectively, similar to the earlier studies.

Complete remission in both the primary tumor and the lymph nodes (ypT0N0) was the best possible pathological outcome of neoadjuvant therapy. Many studies have reported that patients who achieve pathological complete response (PCR) after therapy have higher chances of overall survival.^12^,^13^ Pathological complete response has ranged from 25% to 43% in NACRT studies but was uniformly low in NACT groups ranging from 0% to 3%. In line with previous studies, the PCR rates were 39.6% in the NACRT group and 13.4% in the NACT group, which revealed a trend toward significance (*P*=0.0675).

In the present study, the addition of minimally invasive surgery with better postoperative care may have contributed to favorable surgical outcomes with improved morbidity, no 30-day mortality, and an equivalent oncological outcome. Luketich et al.^14^ retrospectively studied postoperative outcomes in patients undergoing minimally invasive esophagectomy (MIE) by either MIE-neck or a MIE-chest approach. The MIE Ivor Lewis approach, which was associated with reduced recurrent laryngeal nerve injury and mortality of 0.9%, was the preferred approach. The median length of hospital stay (8 days) and ICU stay (2 days) were similar between the two approaches.^14^ In a meta-analysis comparing minimally invasive esophagectomy and open surgery, Hanna et al. found the disease-free and overall survival rates to be similar to those achieved by open surgery.^15^ In a large cohort study, MIE had equivalent overall survival and recurrence-free survival when compared to open esophagectomy.^16^

A significant number of patients (37%) had an unequivocal progression of disease during the course of therapy with NACT in our study. In line with the results of other studies, the resectability rates in the NACRT group was 94%.

The difference in resectability in the present study in the NACRT group can clearly be attributed to the improved response of the tumor to weekly concurrent chemoradiotherapy, as seen by better tumor regression scores and a significantly smaller tumor size as compared to the NACT group. There was a worsening and progression of disease status in the NACT group as evidenced by poorer TRG scores and a larger median residual tumor size.

Usually, patients with adenocarcinoma and those with gastroesophageal junctional tumor and subcardial tumors tend to have higher rates of unresectable disease and reduced responses to preoperative therapy. This may be due to the presence of subclinical serosal disease not detectable by present imaging modalities. The use of diagnostic laparoscopy in pre-therapy staging can potentially mitigate this issue, thereby appropriately staging patients.

The complication rates and mortality in this series appears to be similar to that reported in contemporary series like the NeoRes trial.^17^

Whether esophageal and esophagogastric junction tumors should be treated with preoperative chemoradiotherapy or with perioperative chemotherapy, as suggested by the available evidence, is still unclear. The CROSS trial, which included esophageal and gastroesophageal tumors, has clearly shown that NACRT improves overall survival and disease-free survival over surgery alone.^4^ In the German POET trial, where patients with esophagogastric-junction tumors only were enrolled and randomly assigned to preoperative chemotherapy or chemoradiotherapy, there was a significantly higher probability of showing a pathologically complete response (15.6% versus 2.0%) or tumor-free lymph nodes (64.4% versus 37.7%) at resection in favor of preoperative chemoradiotherapy. Preoperative chemoradiation therapy also improved the 3-year survival rate from 27.7% to 47.4%.^10^ The Scandinavian NeoRes trial comparing postoperative morbidity and mortality after NACT and NACRT found no significant difference in the incidence of complications between patients randomized to NACT and NACRT; instead, the complications were more severe in their NACRT group.^17^ The long-term outcomes of this trial have not yet been reported. The present study demonstrated better rates of PCR, smaller tumor size, complete tumor regression (TRG 0 score), and resectability in favor of the NACRT group, in line with other studies. Randomized studies with a larger patient cohort are required to answer this question.

## CONCLUSIONS

In resectable carcinoma of the esophagus patients, the R0 resection rates and short-term surgical morbidity rates were similar between both preoperative chemotherapy and preoperative chemoradiotherapy groups. There was a significant increase in resectability rates and histopathological response rates with a trend toward increased pathological complete response in the preoperative chemoradiotherapy group. However, this small single-center study has the risks of bias. Adequately powered larger multicentric randomized studies are required to study the relative efficacy in both groups.

## Abbreviations

AUC: area under the curve
BMI: body mass index
BSA: body surface area
ECOG PS: Eastern Cooperative Oncology Group performance status scale
ECOG: Eastern Cooperative Oncology Group
GTV: gross tumor volume
ICU: intensive care unit
LVI: lymphovascular invasion
NACRT: neoadjuvant chemoradiotherapy
NACT: neoadjuvant chemotherapy
PCR: pathological complete response
PNI: perineural invasion
R0 resection: microscopically margin-negative resection
R1 resection: removal of all macroscopic disease, but microscopic margins are positive for tumor
THE: transhiatal esophagectomy
TRG: tumor regression grading
TTE: transthoracic esophagectomy.
